# How Does Birth Order and Number of Siblings Affect Fertility? A Within-Family Comparison Using Swedish Register Data

**DOI:** 10.1007/s10680-019-09525-0

**Published:** 2019-04-30

**Authors:** Kathrin Morosow, Martin Kolk

**Affiliations:** 1 Stockholm University Demography Unit, Department of Sociology, Stockholm University, 106 91 Stockholm, Sweden; 2 Stockholm University Centre for Cultural Evolution, Institute for Future Studies, Stockholm, Sweden

**Keywords:** Fertility, Birth order, Intergenerational transmission of fertility, Sweden, Sibling comparison

## Abstract

This study examines how the sibling constellation in childhood is associated with later fertility behaviour of men and women in Sweden. Administrative register data are used to investigate how birth order affects completed fertility, how the number of siblings and birth order jointly affect completed fertility, and in both cases if there are gender differences in these relationships. Our data consist of all fully biologically related siblings in Sweden whose mothers were born between 1915 and 1935 (the younger generation is born primarily in the 1940s, 1950s and 1960s; *N* = 1,472,813). To study the direct effect of birth order on fertility, sibling comparison models are applied, while to analyse the joint effect of number of siblings and birth order, the sample was stratified by birth order. Results show that higher birth order has a negative effect on completed fertility for women; hence, earlier-born women show overall higher fertility than later-born women. Parity transitions indicate that later-born women are less likely to have two or more children, while no overall gradient for men can be found. The number of siblings is more positively associated with completed fertility for firstborn than for later-born individuals. We conclude that the position in the family of origin can be seen as an additional factor that influences fertility, although effect sizes are rather small.

## Introduction

Research consistently found that the family background has important implications for future childbearing patterns. Both the family composition during childhood and the values acquired from parents have been linked to subsequent fertility behaviour and attitudes (Barber 2001; Cherlin et al. 1995; Duncan et al. 1965; Galster et al. 2007; Thornton 1980). Such family background influences can be both direct and indirect. A consistent finding in demographic research is that the family size of origin is correlated with men’s and women’s own fertility later in life (Anderton et al. 1987; Murphy 1999; Murphy and Knudsen 2002; Murphy and Wang 2001), and it has been suggested that socialization plays an important role in explaining this correlation (Barber 2000, 2001). Parental socio-economic background on the other hand can influence their children’s life course, including fertility patterns, indirectly through opportunity structures (Bengtson 1975).

Thus, structural factors in the family of upbringing—such as number of siblings and the position among them—may have important implications later in life (Kolk 2014a; Murphy 1999), due to siblings getting a different share of a limited pool of parental resources (Blake 1981; Hertwig et al. 2002) or sibling rivalry (Sulloway 1996). Sociologists and psychologists have also suggested that socialization processes or opportunity structures work differently in families of different sizes and that they may differ by birth order of the child (Dunn 2007; McHale et al. 2012).

In this study, we focus on how the sibling composition in the family of origin affects their future fertility, focusing on both an individual’s order in the sibling set, and how the birth order interacts with the number of siblings in the family. Birth order research on outcomes such as education and IQ has a long history going back to the 1930s (see Ernst and Angst 1983; Heer 1986; Steelman et al. 2002). Much early research on birth order effects did not yield many positive results and highlighted the rigorous data requirements for such studies (Ernst and Angst 1983). However, these studies mainly used between-family comparisons, in which children from different families are compared, instead of within-family comparisons, in which only siblings are compared to each other. Additionally, many of these studies did not control for family size and/or used reported information on siblings, which all introduced different kinds of bias to this research (Blake 1989). More recently, researchers have regained interest in birth order and its association with a large variety of outcomes such as health, education, non-cognitive skills or IQ and found support for such effects using sibling comparisons (Barclay and Myrskylä 2014; Barclay and Kolk 2015; Black et al. 2005, 2011; Härkönen 2014; Kalmijn and Kraaykamp 2005). Hitherto, no studies have focused primarily on birth order effects on fertility outcomes, let alone using a within-family approach to isolate birth order effects on fertility patterns. Since birth order directly relates to the experience of upbringing in a family, it is very plausible that there is a relationship between birth order and later fertility outcomes.

More specific, this study aims to answer two questions within the interplay of birth order and sibling size: first, the net effect of birth order on fertility is estimated, avoiding confounding from characteristics that are shared within the family of origin. Secondly, we examine the joint effect of birth order and number of siblings by analysing birth order effects in intergenerational transmission of fertility. Intergenerational transmission of completed fertility means that high parental fertility (number of siblings) is associated with high fertility among their children. Theories on birth order and childhood development suggest that the relative role of parents’ impact on preferences will vary across birth orders, which was one of the central concerns in research on intergenerational transmission of fertility in the 1960s and 1970s (Hendershot 1969; Johnson and Stokes 1976). However, these studies were not able to use very large representative datasets, and more recent studies showed only limited interest in birth order and intergenerational transmission of fertility (with a few exceptions: Booth and Kee 2009; Murphy and Knudsen 2002, 2009).

We use Swedish register data and base our analysis on fully biologically related siblings in Sweden whose mothers were born between 1915 and 1935. This study makes three contributions to research on birth order, fertility and intergenerational transmission. First, we study how the position in the family shapes later fertility outcomes instead of socio-economic or health outcomes. Secondly, by applying a within-family comparison based on a unique sibling identifier we examine whether there is a relationship between birth order and fertility. Using a within-family comparison approach allows us to rule out many potential confounding factors that vary between families, such as SES and all other constant unobserved characteristics in the family of origin. Thirdly, we focus specifically on the interplay of birth order and number of siblings. In the following two sections, we give an overview on how and why birth order may matter for fertility and intergenerational transmission of fertility, before proceeding with a description of the data, methods and results.

## Why Birth Order Matters for Fertility—Background and Previous Research

There are various reasons to assume that social and psychological mechanisms within families may relate to birth order effects in fertility. One likely pathway for birth order effects on fertility is related to birth order effects on socio-economic outcomes, as it is known that, for example, education and income in many contexts are connected to fertility. Earlier research using between-family designs found only ambiguous support for birth order effects on socio-economic outcomes, while recent studies using models that isolate a more “causal” effect of birth order by comparing only siblings have consistently shown positive associations between several socio-economic outcomes and early birth order (Black et al. 2005; Kalmijn and Kraaykamp 2005). These studies found that lower birth order leads to higher educational attainment (Barclay 2015; Black et al. 2005; De Haan 2010; Kantarevic and Mechoulan 2006) as well as higher educational aspiration (Bu 2014). Black et al. (2005) also find that early birth order is associated with earnings and full-time employment, especially for women. An important explanation for why earlier-born siblings cope better is that parents’ resources (including non-economic resources such as time) are fixed, and consequently, having more children leads to fewer available resources per child (Blake 1989). This dilution of parental resources results in a cumulative advantage of earlier-born children over later-born children, and explains their favourable outcomes (Hertwig et al. 2002). Another explanation for birth order effects is Zajonc and Markus’ (1975) confluence model of intellectual development. They argue that firstborn children develop cognitively at a quicker pace due to higher intellectual stimulation. Firstborn solely interact with parents until siblings are born, while later-born children always interact with both parents and other children. Additionally, health researchers have considered physiological explanations for worse outcomes of later born, such as higher incidences of infections among later born (Barclay 2014).

**Fig. 1:**
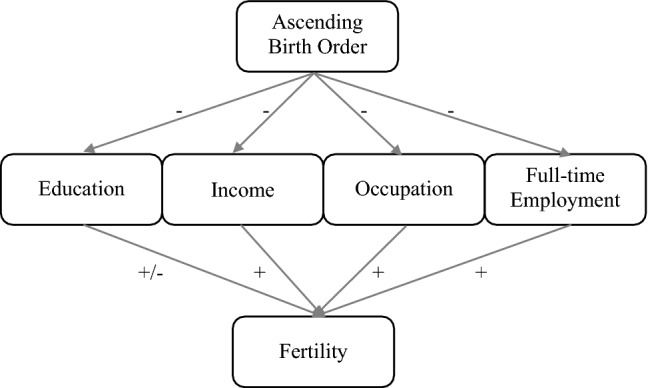
Summary of previous research on how socio-economic factors mediate the relationship between birth order and fertility

These socio-economic factors (that are influenced by birth order) are also important determinants for fertility outcomes, but this relationship varies substantively across context. For Sweden, it has been shown that education is only weakly related to completed fertility (Hoem et al. 2006; Jalovaara et al. 2018), while other status indicators such as employment and income appear to be positively associated with fertility (Andersson 2000; Duvander and Andersson 2003). Income at age 45 seems to be positively associated with fertility for men, whereas there is a weak positive gradient for women (Boschini et al. 2011). In summary, we would expect a weak negative gradient between higher birth order and income and subsequently a positive gradient between income and fertility, to produce a weak negative birth order and fertility gradient, which is likely to be slightly stronger for men than for women. It should be noted, however, that the relationship between education, income and fertility may not be due to direct effects, but that groups with low or high fertility differs on other traits that are correlated with education (Jones et al. 2008; Tropf and Mandemakers 2017). Figure 1 summarizes the literature on birth order effects on fertility through socio-economic outcomes. Overall, being earlier born is assumed to have a positive effect on fertility through socio-economic status advantages.

Both psychological and sociological perspectives focus on how the birth order in the sibling set affects the developmental processes in childhood and offer a second pathway for birth order effects on fertility. Siblings share many important influences that determine their development and later life outcomes, such as parental socio-economic background, family stability, neighbourhood, and—largely—genes. However, despite their common background siblings still differ considerably. Some of these factors are independent of birth order (e.g. genetics), while processes such as socialization may be affected by birth order. If parents treat and socialize firstborn children different from later-born children, this would imply different conditions during upbringing. Individual childbearing behaviour and preferences are, amongst others, results of socialization. However, little research has focused on the structural characteristics, such as if the number of siblings and birth order affects socialization of fertility preferences (Steelman et al. 2002). However, the degree of family orientation may depend on both the number of siblings (Booth and Kee 2009; Murphy 1999) and birth order. Older siblings may have a more caretaking role towards younger siblings and therefore develop more family-oriented preferences. On the other hand, younger siblings are exposed to a larger family for longer parts of their childhood, which could lead to a high family orientation as well. Such factors might also vary by gender: Cools and Hart (2017) suggest that women with more siblings might reduce later childbearing, as they observe the strains of childbearing their mothers’ face, while men might not show the same response. Therefore, from a perspective of how family composition affects socialization, it is unclear whether earlier-born siblings would develop more family-oriented preferences or the opposite.

Early psychoanalytical research on personality traits gives another possible pathway on how birth order could affect fertility, as personality traits have been shown to be associated with both fertility and birth order. Adler and his theory of individual psychology was the original advocate of this line of research (Adler 1928; Freese et al. 1999; Whiteman et al. 2011). Adler suggests that sibling relationships are important for personality development and that the transition from being an only child to having siblings causes firstborn children to conform stronger to their parents (Adler 1928). In order to lower competition and rivalry for parent’s attention and resources, siblings eventually differentiate into different niches within the family and develop different personality traits (Whiteman et al. 2011). Sulloway’s family-niche model builds on Adler’s theory and predicts that earlier-born siblings are expected to be more conservative and later born more rebellious (Sulloway 1996).

A number of studies examined how birth order is related to the Big 5 personality traits (openness, conscientiousness, extraversion, agreeableness, and neuroticism) (Ernst and Angst 1983). Several studies using a between-family approach did not find a link between birth order and personality (Ernst and Angst 1983; Marini and Kurtz 2011; Rohrer et al. 2015), or contradict Sulloway’s model (Michalski and Shackelford 2002; Pollet et al. 2010). However, studies using a within-family design find firstborn to be more conscientious, achieving and dominant extravert, while second or later born were found to be more rebellious, liberal, agreeable, more sociable and overall extrovert (Beck et al. 2006; Dixon et al. 2008; Healey and Ellis 2007; Paulhus et al. 1999)—with often stronger effects for sisters than for brothers.

**Fig. 2:**
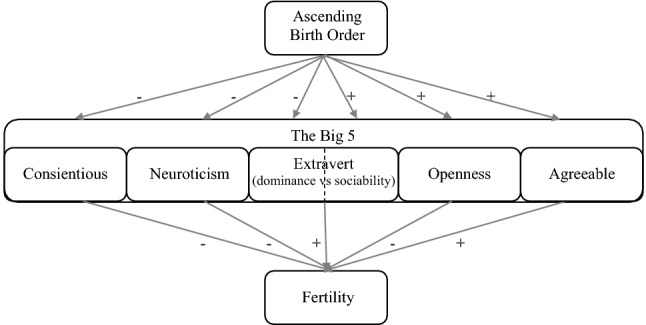
Summary of previous research on how personality traits mediate the relationship between birth order and fertility

Research that examined how the big 5 personality traits are associated with fertility suggests that higher levels of conscientiousness, neuroticism, and openness are related to lower levels of fertility, while extraversion and being agreeable seem to increase fertility (Jokela et al. 2009, 2011; Jokela 2012; Skirbekk and Blekesaune 2014). Figure 2 summarizes previous research on how personality traits may explain the relationship between birth order and fertility. In contrast to the socio-economic status perspective, this literature implies that earlier-born children have lower fertility than later-born children do. Despite the suggested competing effects, however, effect sizes indicate that socio-economic status is a stronger predictor of fertility than personality is.

Previous discussed research has examined whether siblings of the same family differ on outcomes based on their birth order in the family. It is also possible that the extent to which children adopt their parents’ behaviour differs according to birth order. Researchers have therefore examined how number of siblings (parental fertility) is associated with completed fertility and how the effect varies according to the birth order of the child. Number of siblings is a strong and influential predictor of fertility (Kolk 2014a; Murphy 1999). Hence, the joint effect between number of siblings a person grows up with and the birth order of that individual needs to be discussed for later fertility outcomes. Various studies for a large number of societies found intergenerational transmission of completed fertility (Murphy 1999, 2013) and age at first birth (Barber 2001; Dahlberg 2013). This correlation between number of siblings and own fertility was primarily explained through childhood socialization and the transmission of socio-economic status (Barber 2000, 2001; Kolk 2014a, 2015). Studies examining exogenous variation in number of siblings (Kolk 2015; Cools and Hart 2017) have found little effect of number of siblings on fertility, suggesting that intergenerational correlations are explained by shared values and preferences across generations, rather than the causal effect of the number of siblings. Some authors have also suggested that genetics plays a role (Kohler et al. 1999; Tropf and Mandemakers 2017). Childhood circumstances are important for what views, preferences, and behaviours children acquire, but, as mentioned above, these circumstances are not the same for each sibling. If the childhood experience differs substantively by birth order, it is reasonable to expect that this will result in differences in intergenerational transmission by birth order.

In the 1960s and 1970s, research on intergenerational transmission of fertility used birth order as one of the key explanatory variables (Hendershot 1969; Johnson and Stokes 1976; McAllister et al. 1974), largely motivated by theories of higher conformity to parental behaviour and preferences among first or early born as suggested by Adler and Sulloway. This implied that firstborn children show higher fertility when having more siblings than later born; hence, one would find a stronger transmission effect for early-born children. Last-/later-born children are assumed to face less rigorous parenting, which is suggested to make them more carefree and separate themselves by being rebellious. There is some empirical evidence for such a relationship (Schachter 1964). If later-born children oppose their parents’ role model, no or little correlation would be found between number of siblings and own fertility. Therefore, the niche model gives reason to expect earlier-born children to be stronger affected by more siblings than later born.

Intergenerational transmission research on fertility, using birth order mainly as a control in a regression framework, suggests that firstborn children are stronger affected by their family than later-born children (Murphy 1999). Research from the 1960s and 1970s supports this idea, mainly drawing on correlations (Hendershot 1969; Johnson and Stokes 1976; McAllister et al. 1974). More recent studies that look at the effects of family size and birth order find that early-born children are likelier to show stronger intergenerational transmission, while later-born children are more inclined to be part of an intergenerational contrast group (Fasang and Raab 2014). Booth and Kee (2009) show a stronger, but non-significant, relationship between fertility patterns for firstborn children, while Murphy and Knudsen (2002) do not find evidence for this birth order effect on completed fertility.

## Research Design, Data and Methods

This study examines both the isolated effect of birth order on fertility, and how birth order affects the relationship between number of siblings and fertility. However, birth order and family size of origin are interrelated, and a careful approach is necessary to disentangle the different effects.

Previous research paid more attention to the number of siblings as a predictor of family size than to birth order. While several studies find that family size influences own fertility (Kotte and Ludwig 2011; Murphy 1999; Murphy and Wang 2001), birth order and family size are directly connected, as, for example, a fourth born will always grow up in a family of at least four children. Family size is endogenous and closely related to parent’s preferences. Therefore, parents that choose to have many or few children are selected in various ways, and children of these parents may be selected for similar reasons. To solve such issues, we use a within-family comparison analysis for our first research question. Using a sibling fixed-effects regression model compares only siblings to each other instead of individuals between families, which means that shared factors between siblings, that are assumed to be constant, such as parents’ socio-economic status and number of siblings are controlled for and that differences between families cannot bias the results. This is done based on a unique sibling identifier for every sibling group that share the same father and mother. This research design implies that in the sibling comparison analysis by gender, only siblings of the same sex are compared to one another. The birth order covariate is always calculated based on all siblings, however, independent of gender. Hence, while these models omit sibling sets where only one brother and/or one sister is present, these sibling sets are included in the combined models. Supplementarily, we run linear probability models for each parity transition in order to be able to identify birth order differences between the transitions.

The number of siblings cannot be included in these sibling models, however, as the number of siblings is the same for all siblings in a family. Thus, the second research question aims at quantifying the joint effect of number of siblings and birth order. In order to distinguish those two effects, we stratify our population by birth order and run the analysis for each birth order independently. The relationship of number of subsequent siblings after their own birth order (younger siblings) and own fertility is shown. This was chosen as second-born siblings will always have one older sibling, but we are interested in how an increase in family size affects them. We present such results with separate models for each birth order, where the slope in the graph represents the strength of the relationship between number of siblings and own fertility. Ordinary least square (OLS) regression analysis has been used for both research questions, in which number of children of the younger generation is our dependent variable.

We use administrative population registers for the complete population of Sweden. Individuals are connected by means of a personal identity number, allowing accurate linkages across generations inside Sweden. The analysis population consist of all Swedish-born individuals with a mother born between 1915 and 1935 (parental generation). Whereas the younger generation is born between 1932 and 1988 (with almost 99% born between 1937 and 1971) and is observed in 2012, thus prior to applying our sample selection criteria, the data include completed fertility for virtually all members of the younger generation. Although both our older and younger cohorts cover very different historical times, effects would not reflect cohort effects as the cohort fertility rate in Sweden was very stable over these periods (“Appendix”, Fig. 8). Nevertheless, we also control for changes over time by including controls for the birth year of our younger generation. We only look at individuals and their full siblings, in families in which there are no maternal or paternal half- or step-siblings, since the theoretical meaning of (potentially not resident) half- or step-siblings for later life fertility is unclear. Although this sample choice could increase the selectivity of the sample somewhat as complex families become more common over time, we use this more select sample to maximize the internal validity of our results as we make sure that the siblings that are compared spent most of their time in the same household. We also restrict our study population to Swedish-born individuals that were alive or had never out-migrated from Sweden at age 45. Measures on the number of siblings of the younger generation (the fertility of the older generation) and birth order are calculated by means of the Swedish multigenerational register. Information about our study population can be found in Table 1. In “Appendix” Table 2, we present a flow chart on how our different sample restrictions affect our eventual population.

**Table 1:** Descriptive statistics of the population under study

Variable	*N*	%
Fertility younger generation		
0	228,457	16.76
1	192,742	14.14
2	566,861	41.59
3	279,262	20.49
4	71,481	5.24
5	17,162	1.26
6	4668	0.34
7	1425	0.10
8	515	0.04
9	194	0.01
10+	171	0.01
Fertility parental generation/sibship size		
1	150,058	11.01
2	482,835	35.43
3	370,423	27.18
4	191,803	14.07
5	86,219	6.33
6	40,764	2.99
7	20,035	1.47
8	10,063	0.74
9	52	0.38
10+	5538	0.41
Birth order younger generation		
1	593,081	43.51
2	441,935	32.43
3	200,287	14.70
4	77,018	5.65
5	29,169	2.14
6	1191	0.87
7	5168	0.38
8	2324	0.17
9	1071	0.08
10+	975	0.07
Year of birth younger generation		
1932–1936	932	0.68
1937–1941	73,857	5.42
1942–1946	228,881	16.79
1947–1951	317,775	23.32
1952–1956	318,739	23.39
1957–1961	241,389	17.71
1962–1966	129,309	9.49
1967–1971	38,063	2.79
1972–1976	5322	0.39
1977–1981	273	0.02
1982–1988	10	0.00
Year of birth father		
1885–1894	878	0.06
1895–1904	16,673	1.22
1905–1914	262,777	19.28
1915–1924	638,725	46.86
1925–1934	419,522	30.78
1935–1944	24,303	1.78
1945–1951	60	0.00
Year of birth mother		
1915–1924	709,265	52.04
1925–1935	653,673	47.96
Gender		
Men	696,395	51.10
Women	666,543	48.90
Total	1,362,938	100

Swedish register data, authors’ own calculations

In general, very young maternal age is associated with negative child outcomes in education, cognitive abilities and health (Conley 2001; Geronimus et al. 1994), which is why we adjust for maternal age in our models. Similar to family size and birth order, mother’s age and birth order are directly related as later-born children are always born to older mothers. This interrelation could be especially problematic when it comes to the birth order effect on children’s socio-economic outcomes. Assuming that increasing mother’s age has a positive effect on children’s educational outcomes and increasing birth order has a negative effect, we would suggest a counteracting effect on children’s socio-economic status that needs to be controlled for. Furthermore, mother’s age at birth for each sibling functions as a proxy for birth spacing in the fixed-effects approach, which is important as a larger age gap between siblings, is presumed to be associated with lower birth order effects (Beck et al. 2006; Sulloway 1996).

## Results

### Birth Order and Fertility

**Fig. 3:**
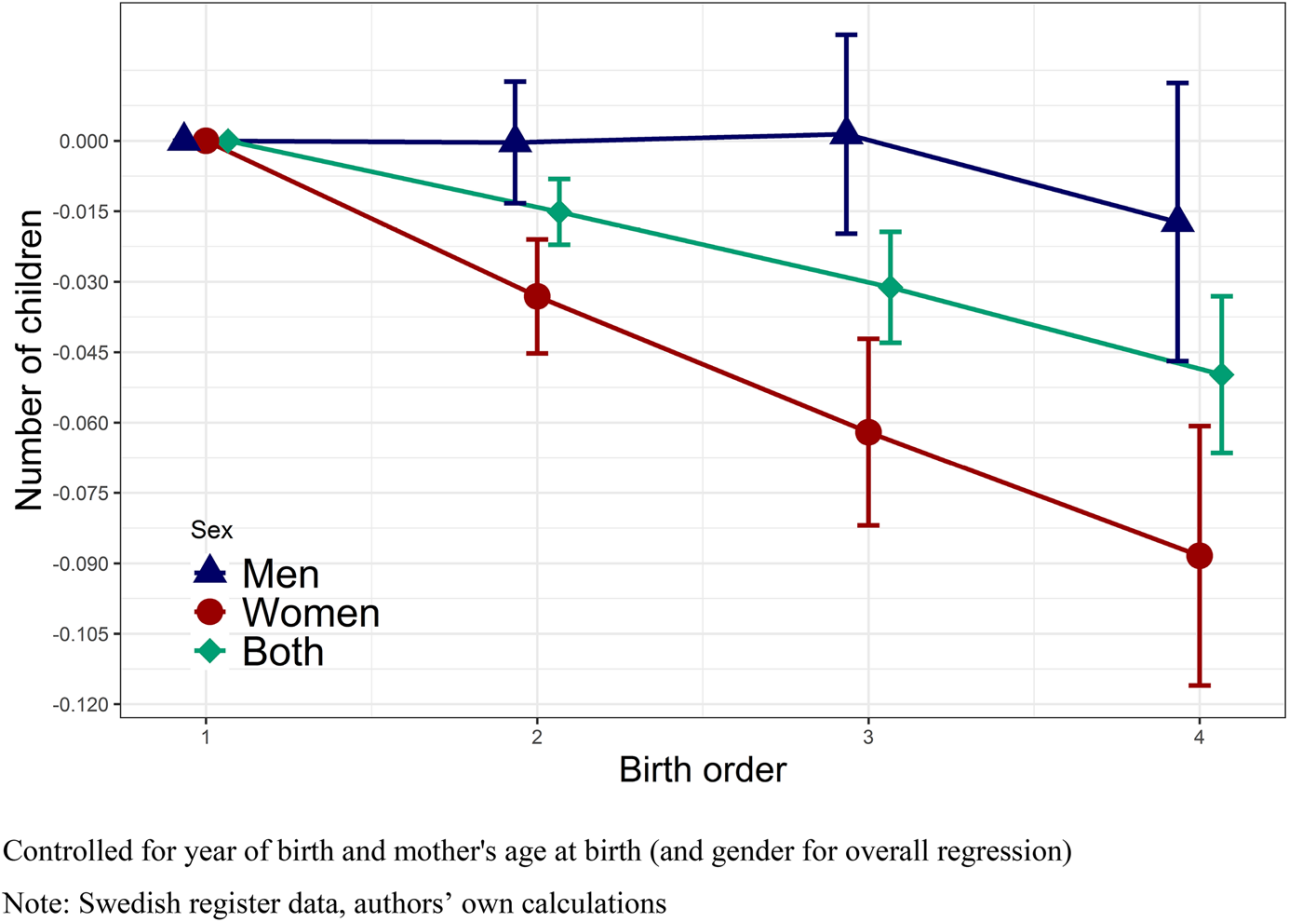
OLS regression on number of children by birth order, sibling fixed-effects models, Swedish-born men and women with a mother born between 1915 and 1935

In order to investigate how birth order affects fertility, we run sibling fixed-effects models. Firstly, Fig. 3 shows the results of the OLS sibling comparison regression with number of children as the dependent variable; the full table can be found in “Appendix” (Table 3). We restrict the presented results to birth order 4 and below, as a larger birth order represents a rather small fraction of all observed individuals, though we present results for higher birth orders in the appendices. Overall, the effect of birth orders 5 and 6 shows exaggerated effects of what we show for birth order 4. Results show that higher birth order is associated with significantly fewer children for women. The effect follows a monotonic decrease with later-born sisters having fewer children compared to their firstborn sister. For the completed fertility of men, on the other hand, the effects are close to zero for all birth orders, with only a small non-significant negative effect for birth order 4 and higher. The difference between a firstborn and fourth-born woman (0.09 fewer children) is equivalent to a change in 0.04 standard deviations in fertility for our outcome variable. (For birth order 6, the effect is 0.13 fewer children.) Thus, the results suggest that birth order shows a negative relationship for women, with more children amongst early-born sisters than later-born sisters, though the effects are not very large in substantive terms. No clear association between birth order and fertility can be found for men, however. We also ran analyses for both sexes comparing all siblings, and including a covariate for sex, which predictably shows results in between those presented for men and women. When using between-family comparisons for this analysis, a strong positive effect of birth order on fertility is found for both men and women, illustrating that a within-family approach is necessary to capture birth order effects that are not biased by selection or between-family differences (see Fig. 9).

**Fig. 4:**
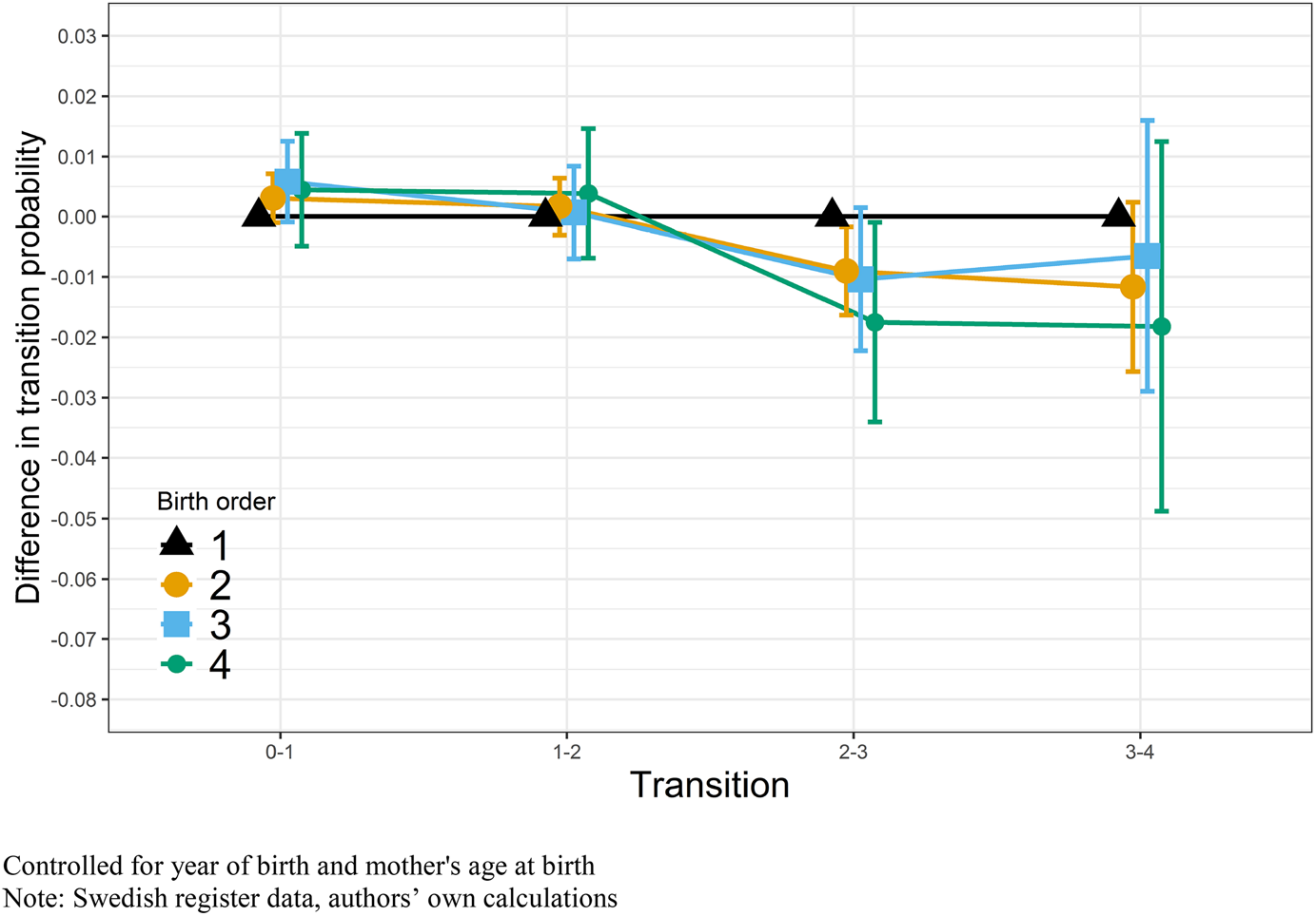
OLS regression on transitions to different parities by birth order, sibling fixed-effects models, Swedish-born men with a mother born between 1915 and 1935

In a next step, the analysis was extended to examine transitions to different parities by birth order. Focusing on each parity transition enables us to show nonlinear relationships between a person’s birth order and the distribution of their eventual number of children. Figure 4 depicts these results for men from the sibling fixed-effects models and shows different birth order effects for brother’s parity transitions. In line with results for completed fertility, we find no overall statistically significant association, but our estimates indicate that later borns are slightly more likely to have a first child, while less likely to have three or more children, though most of these estimates are imprecise and not statistically significant.

**Fig. 5:**
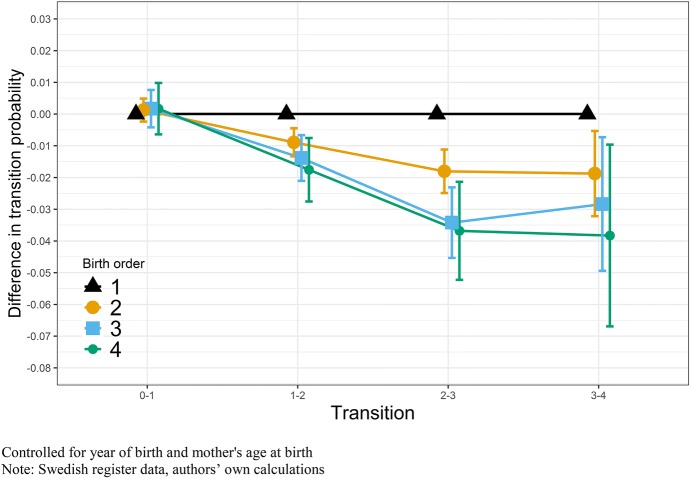
OLS regression on transitions to different parities by birth order, sibling fixed-effects models, Swedish-born women with a mother born between 1915 and 1935

Figure 5 illustrates this relationship for women; the results show a pattern of decreasing fertility by higher birth order seen in the previous model for completed fertility. For entry to parenthood, we find a weak pattern of firstborn women to have lower transition rates compared to their later-born sisters; this, however, is not significant. Hence, sisters of different birth orders do not differ in their likelihood to become a mother. For transitions to higher order births, we find the opposite pattern with early-born sisters having higher transition probabilities. This relationship is significant for the second, third, and fourth parity transitions, but not for the highest parities due to the rarity of these events. The overall negative association between higher birth order and number of children for women seen in Fig. 3, therefore, consists of a substantial negative influence of birth order on parity transitions two to four. Summarizing, we find few effects for men. For women, the negative effect of birth order is more prominent at parities beyond one. The tables to these models can be found in “Appendix”, including additional parity transitions and birth orders (Tables 4, 5).

### Birth Order, Number of Siblings and Fertility

The combined effect of both number of siblings and birth order on fertility is examined in the second part of the analysis. Theoretical approaches and research suggest that the influence of parental characteristics, such as number of siblings, on their children might be larger for early-born siblings. Our results are consistent with the previous research, showing that intergenerational transmission of completed fertility exists in Sweden (Dahlberg 2013; Kolk 2014a). A positive effect of family size of origin can be seen for individuals childbearing (Pearson Correlation: 0.1027; Pearson Correlation with controls for year of birth and mother’s age at birth: 0.1065). Results, for men and women, showing the association between parents’ fertility and their children’s completed fertility are shown in “Appendix” (Fig. 10). Independent of birth order, women seem to be stronger affected by their parents’ fertility (or put differently, number of siblings) than men, which is in line with the previous research (Murphy 1999).

**Fig. 6:**
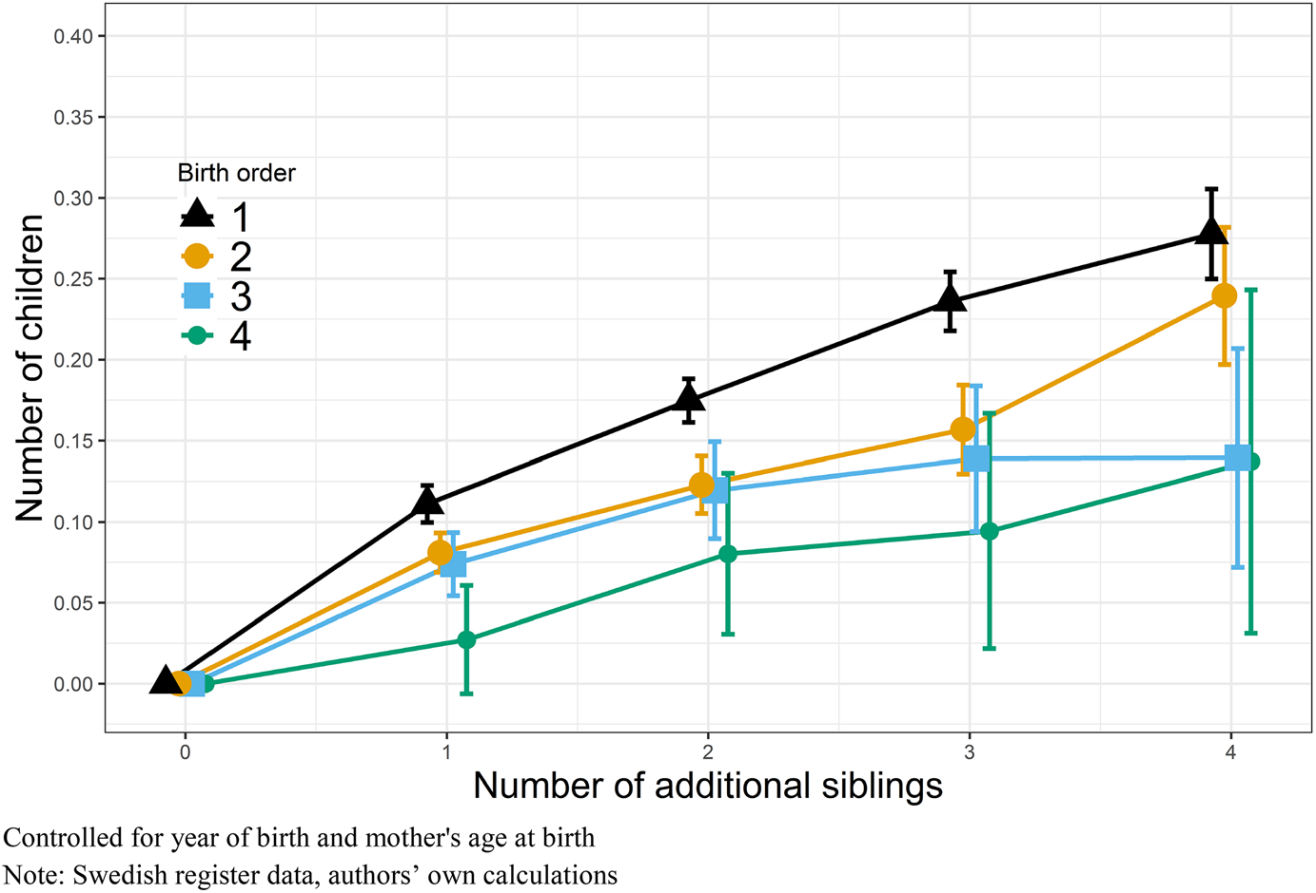
OLS regressions on number of children by additional siblings, stratified by birth order, Swedish-born men with a mother born between 1915 and 1935

**Fig. 7:**
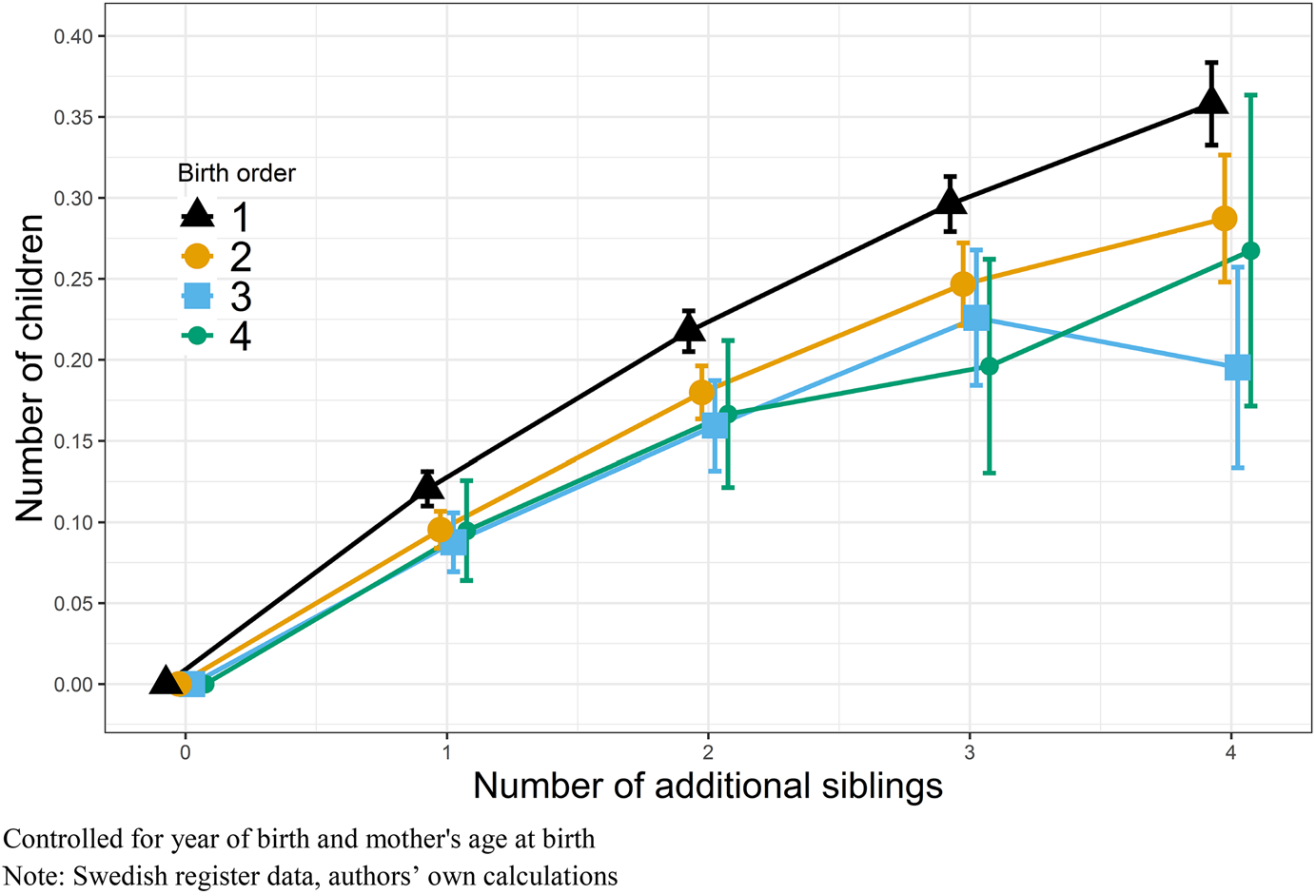
OLS regressions on number of children by additional siblings, stratified by birth order, Swedish-born women with a mother born between 1915 and 1935

**Fig. 8:**
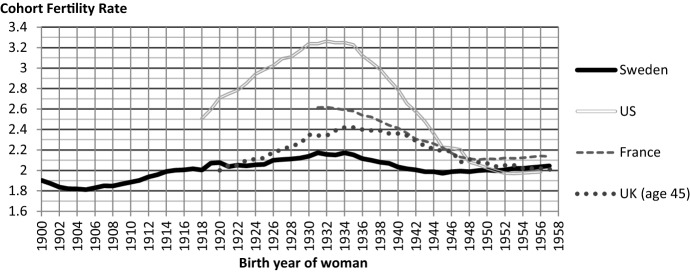
Cohort fertility rate in Sweden, France, the USA and the UK, 1900–1958. *Source*: Kolk (2014b)

**Fig. 9:**
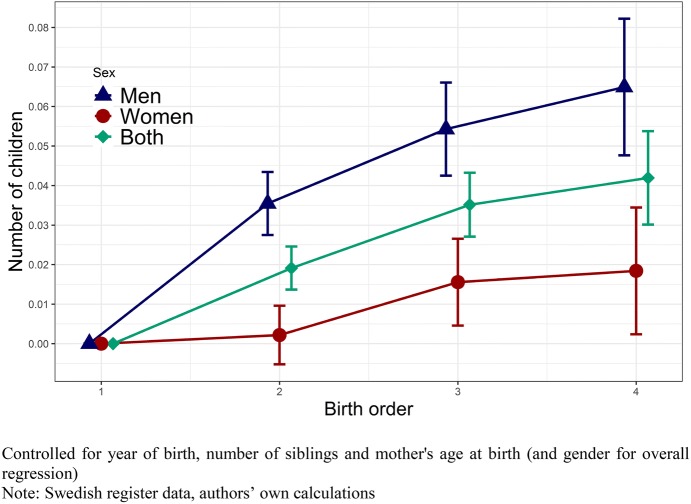
OLS regression on number of children by birth order, non-fixed effects, Swedish men and women born in 1932–1988

**Fig. 10:**
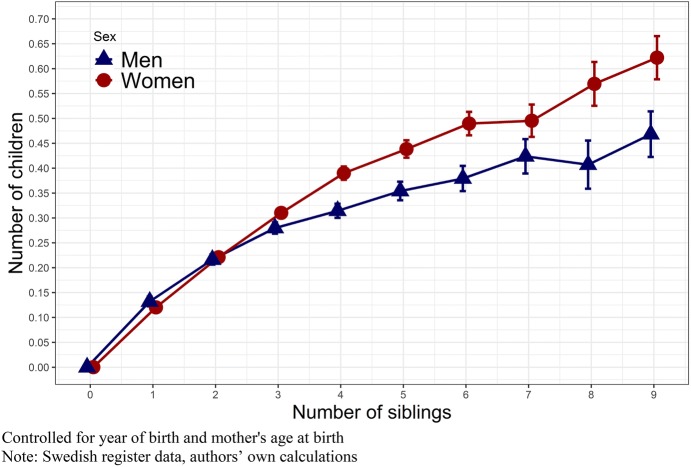
Number of children by number of siblings for men and women relative only children, Swedish-born men and women with a mother born between 1915 and 1935

To identify whether the fertility of firstborn individuals is stronger related to the number of siblings, than the fertility of later-born individuals, we stratify our population by birth order and examine subsequent siblings born after the index person, which means younger siblings. Figures 6 and 7 show the effect of additional siblings on fertility for men and women, respectively (Tables can be found in “Appendix”: Tables 6, 7). For men and women of all birth orders, we find a clear relationship where additional siblings are associated with higher own fertility. One additional sibling for a firstborn means the effect of having one sibling, while an additional sibling for a second born means a total number of three siblings. In other words, additional siblings here are younger siblings. For all firstborn men (Fig. 6), we find that if they grow up with an additional sibling their own fertility increases by 0.11 children, with each additional sibling after that eventual fertility increases by additional 0.06 children. For later-born men, the increase in fertility, by number of siblings, is smaller, suggesting that later borns are less impacted by family size of origin (and their parents’ fertility preferences). For women (Fig. 7), we find a largely similar pattern, where firstborn women’s fertility is stronger related to their parent’s childbearing, than later-born women’s. Firstborn women with one additional sibling show an increase in own fertility by 0.12 children, two additional siblings increases their own fertility by 0.22 children, and three younger siblings increases their own fertility by 0.30 children. For later born, one additional sibling increases the fertility by about 0.10 children, and subsequent siblings by additional 0.07 children. Overall, the effect of number of siblings is a stronger predictor of own fertility for women, but we see slightly smaller differences by birth order, even if parental behaviour also in this case appears more important for women.

Overall, we find evidence that firstborn men and women show higher intergenerational transmission of fertility, with lower levels for higher birth orders. Our results therefore largely conform to theories of firstborn being more affected by parental behaviour, and potentially more compliant than later-born siblings, and this suggests that intergenerational transmission is linked to the number of younger siblings, rather than just siblings per se. Our birth order differences are most clear when contrasting the firstborn with everyone else, rather than finding a gradual decrease in the effect of parental behaviour at each subsequent birth order. The results presented in Figs. 6 and 7 are robust across different model specifications, including models with total number of siblings.

## Conclusion

We have examined two aspects of how birth order is affecting fertility outcomes; firstly, we examined the direct effect of birth order on fertility, and secondly, the interplay of birth order and number of siblings on fertility. By applying sibling comparison models, we are able to control for all shared factors between siblings including unobserved heterogeneity and the fact that higher birth order siblings come from bigger families. These sibling fixed-effects OLS models show a negative effect of increasing birth order on completed fertility for women, while no substantive association can be found for men, though all effects are rather small. Birth order effects on parity transitions show that for women higher birth order has a negative effect on the second, third, and fourth parity transitions. Hence, later-born women are less likely to have two or more children compared to their firstborn sisters. Being a later-born sibling for man is associated with a lower likelihood to have three or more children, but effects are much smaller. Overall, these results indicate that firstborn men and women are less likely to have only one child and more likely to have a higher number of children compared to their brothers and sisters. For women, this translates into lower completed fertility of later-born sisters. The largest effect sizes are found for parity progression ratios for later-born women, who typically are up to 5 more percentage points less likely to have higher order births than firstborn women. Nevertheless, comparing the effect sizes to other well-known fertility predictors, birth order seems to be a less important factor.

Theoretically, the resource dilution hypothesis as well as the confluence hypothesis led to the assumption that earlier-born individuals may have higher completed fertility, given the positive association between status and fertility in Sweden. On the other hand, previous studies from psychological research on personality and birth order would suggest a negative effect of being earlier born on fertility outcomes. Our results for women show that the effect of birth order on total family size is consistent with explanations of lower socio-economic status with higher birth order, which may lead to lower fertility. However, we do not find such a pattern for men, which is not consistent with a socioeconomic status based explanation, especially when socioeconomic indicators are more strongly linked to fertility for men in Sweden (Boschini et al. 2011; Jalovaara et al. 2018). Instead, our results on a negative association between increasing birth order and fertility for women seem more consistent with socialization theory. Early-born women that grow up with younger siblings seem to have a higher fertility than their younger sisters. This higher fertility for early-born women, but not men, is consistent with gendered expectations on part of the parents in which firstborn women face stronger parental encouragement or pressure to establish a family. Such pressure might be smaller for later-born daughters if their sisters had a child already. Another possible explanation is that this result merely reflects higher family orientation among women that grow up with younger siblings, though no similar pattern is found for men. These findings might also be consistent with early ideas that suggest that firstborn women adopt traditional female gender roles to a higher degree than later-born women, and with it unusual high fertility (Kammeyer 1966).

What role does the number of siblings play in combination with birth order? Intergenerational transmission of family size has previously been shown, but does the importance of the number of siblings differ by birth order? Birth order and socialization theory suggest a stronger intergenerational resemblance for firstborn children, but previous studies did yield mixed results. Stratifying the population by birth order identifies different effects of number of siblings on earlier- and later-born individuals. We find that firstborn men and women show higher levels of fertility, as well as increasing fertility with higher numbers of siblings. Few significant differences can be found among second or higher birth orders, except for their lower transmission compared to firstborn. Accordingly, our results are consistent with previous research on intergenerational transmission of fertility that has found firstborns to show higher fertility (Booth and Kee 2009; Hendershot 1969; Johnson and Stokes 1976; Murphy and Knudsen 2002).

Overall, this shows that firstborn siblings show a higher fertility within their family, as well as with increasing number of siblings. Finding a negative relationship between increasing birth order and fertility for women complements other research that has recently found birth order effects for various later life outcomes (Barclay and Myrskylä 2014; Barclay and Kolk 2015; Black et al. 2005). Our results also highlight the necessity of a within-family approach as the between-family association is reversed when fixed-effects models are applied. Birth order is related to the childhood environment in the family of origin, and it is perhaps not surprising that such experiences are related to later decisions about family formation. We note that the effects do not differ for the decision to become a parent, but for decisions to have a comparatively large family, as well as that our effects are more pronounced for women. As such, our research is useful as a comparison point between research in psychology focusing on personality and preferences, and research looking at quantitative outcomes in adulthood. Further research should aim to understand why birth order in childhood appears to affect decisions about family formation in adulthood. By collecting information on childbearing preferences and ideals among men and women of different birth orders, it would be possible to better understand why later-born women have fewer children and are less affected by younger siblings.

## Appendix

See Figs. 8, 9, 10 and Tables 2, 3, 4, 5, 6, 7.

**Table 2:** Sample restrictions

Exclusion criteria	Men		Women		Total
	*N*	*N* excluded	*N*	*N* excluded	*N*
Birth order effects on fertility (fixed-effects OLS)					
Total in Swedish registers with a mother born between 1915 and 1935 and parent ID’s known	1,066,370		1,014,569		2,080,939
Swedish born (mother, father, index person)	880,465	185,905	840,790	173,779	1,721,255
Never migrated at age 45	869,332	11,133	824,837	15,953	1,694,169
Alive by age 45	848,645	20,687	814,077	10,760	1,662,722
No half siblings	696,395	152,250	666,543	147,534	1,362,938
No only children	620,203	76,192	592,677	73,866	**1,212,880**
Birth order effects on intergenerational transmission of fertility (OLS)					
Total in Swedish registers with a mother born between 1915 and 1935 and parent ID’s known	1,066,370		1,014,569		2,080,939
Swedish born (mother, father, index person)	880,465	185,905	840,790	173,779	1,721,255
Never migrated at age 45	869,332	11,133	824,837	15,953	1,694,169
Alive by age 45	848,645	20,687	814,077	10,760	1,662,722
No half siblings	696,395	152,250	666,543	147,534	**1,362,938**

Bold numbers reflect the final sample for the two analyses

**Table 3:** OLS regression on number of children by birth order, sibling fixed-effects models, Swedish men and women born in 1932–1988

Variables	All	Men	Women
Birth order			
1	0	0	0
2	− 0.015*** (0.004)	− 0.000 (0.006)	− 0.033*** (0.006)
3	− 0.031*** (0.006)	0.001 (0.010)	− 0.062*** (0.010)
4	− 0.050*** (0.008)	− 0.017 (0.015)	− 0.088*** (0.014)
5	− 0.066*** (0.011)	− 0.033* (0.020)	− 0.103*** (0.019)
6	− 0.064*** (0.015)	− 0.024 (0.025)	− 0.133*** (0.023)
7	− 0.068*** (0.021)	0.034 (0.033)	− 0.164*** (0.031)
8	− 0.089*** (0.028)	− 0.067 (0.045)	− 0.120*** (0.042)
9	− 0.037 (0.040)	0.012 (0.064)	− 0.109* (0.056)
10	− 0.139*** (0.044)	0.056 (0.069)	− 0.305*** (0.065)
Year of birth			
1932–1936	0	0	0
1937–1941	− 0.025 (0.018)	0.010 (0.032)	− 0.054* (0.029)
1942–1946	− 0.026 (0.019)	0.018 (0.034)	− 0.052* (0.031)
1947–1951	0.011 (0.021)	0.075** (0.038)	− 0.021 (0.034)
1952–1956	0.058** (0.023)	0.123*** (0.043)	0.035 (0.040)
1957–1961	0.073*** (0.027)	0.133*** (0.049)	0.082* (0.044)
1962–1966	0.028 (0.030)	0.082 (0.055)	0.039 (0.051)
1967–1971	− 0.129*** (0.034)	− 0.076 (0.062)	− 0.088 (0.058)
1972–1976	− 0.550*** (0.043)	− 0.476*** (0.077)	− 0.503*** (0.072)
1977–1981	− 1.005*** (0.102)	− 0.643*** (0.182)	− 1.235*** (0.170)
1982–1988	− 1.781*** (0.427)	− 2.089** (0.852)	− 1.171* (0.606)
Mother’s age at birth			
13–16	0.172*** (0.052)	0.078 (0.093)	0.167* (0.087)
17–18	0.120*** (0.015)	0.188*** (0.027)	0.063** (0.024)
19–20	0.090*** (0.009)	0.126*** (0.017)	0.062*** (0.016)
21–22	0.052*** (0.007)	0.083*** (0.013)	0.038*** (0.012)
23–24	0.027*** (0.005)	0.038*** (0.010)	0.020** (0.009)
25–26	0	0	0
27–28	− 0.018*** (0.005)	− 0.032*** (0.010)	− 0.017* (0.009)
29–30	− 0.041*** (0.006)	− 0.079*** (0.011)	− 0.032*** (0.010)
31–32	− 0.072*** (0.008)	− 0.119*** (0.015)	− 0.042*** (0.014)
33–34	− 0.093*** (0.010)	− 0.155*** (0.018)	− 0.072*** (0.017)
35–36	− 0.122*** (0.012)	− 0.197*** (0.021)	− 0.086*** (0.020)
37–38	− 0.161*** (0.014)	− 0.247*** (0.026)	− 0.146*** (0.024)
39–40	− 0.184*** (0.016)	− 0.297*** (0.030)	− 0.129*** (0.028)
41–42	− 0.229*** (0.020)	− 0.344*** (0.036)	− 0.168*** (0.034)
43–44	− 0.256*** (0.026)	− 0.375*** (0.047)	− 0.187*** (0.044)
45–46	− 0.293*** (0.042)	− 0.403*** (0.075)	− 0.212*** (0.067)
47–48	− 0.238*** (0.091)	− 0.246 (0.150)	− 0.263* (0.155)
49–53	− 0.026 (0.236)	− 0.023 (0.382)	− 0.004 (0.435)
Sex			
Male	0		
Female	0.147*** (0.002)		
Constant	1.87052*** (0.02167)	1.82223*** (0.03930)	2.03909*** (0.03594)
Observations	1,212,880	620,203	592,677
*R*-squared	0.01103	0.00948	0.00683
Number of sibling sets	457,063	374,489	365,730

Swedish register data, authors’ own calculations

Standard errors in parentheses

****p* < 0.01; ***p* < 0.05; **p* < 0.1

**Table 4:** Linear probability models for parity transitions, sibling fixed effects, robust standard errors, Swedish men born in 1932–1988

Variables	Men					
	Transition 0–1	Transition 1–2	Transition 2–3	Transition 3–4	Transition 4–5	Transition 5–6
Birth order						
1	0	0	0	0	0	0
2	0.00306 (0.00208)	0.00168 (0.00241)	− 0.00902** (0.00372)	− 0.01167 (0.00717)	0.02881 (0.02027)	− 0.01460 (0.05079)
3	0.00579* (0.00341)	0.00068 (0.00393)	− 0.01040* (0.00605)	− 0.00652 (0.01147)	− 0.00750 (0.03085)	0.01311 (0.07367)
4	0.00446 (0.00476)	0.00385 (0.00548)	− 0.01751** (0.00844)	− 0.01819 (0.01561)	− 0.00662 (0.04219)	− 0.03060 (0.09951)
5	0.00687 (0.00628)	− 0.00431 (0.00721)	− 0.01756 (0.01106)	− 0.01841 (0.02020)	0.00007 (0.05231)	− 0.00494 (0.12965)
6	0.01438* (0.00820)	0.00018 (0.00937)	− 0.01664 (0.01441)	− 0.02372 (0.02585)	− 0.02852 (0.06330)	− 0.03877 (0.14364)
7	0.01761 (0.01093)	0.01842 (0.01187)	0.01472 (0.01864)	− 0.02682 (0.03167)	0.04627 (0.07373)	− 0.08414 (0.16776)
8	0.03064** (0.01486)	− 0.00838 (0.01647)	− 0.02193 (0.02479)	− 0.05101 (0.04097)	− 0.05051 (0.08975)	− 0.10214 (0.22410)
9	0.01315 (0.02104)	0.02333 (0.02353)	0.02097 (0.03320)	0.07062 (0.05698)	− 0.01873 (0.12826)	0.02650 (0.34402)
10	0.02223 (0.02388)	0.03239 (0.02539)	0.02197 (0.03759)	0.05803 (0.05952)	0.06160 (0.13756)	− 0.66311** (0.26534)
Year of birth						
1932–1936	0	0	0	0	0	0
1937–1941	0.01836** (0.00915)	− 0.00588 (0.01114)	− 0.02775 (0.01752)	− 0.01340 (0.03201)	− 0.03412 (0.08505)	0.19876 (0.24290)
1942–1946	0.02721*** (0.00995)	− 0.01319 (0.01209)	− 0.03351* (0.01887)	0.00664 (0.03489)	− 0.08512 (0.09450)	0.39441 (0.27053)
1947–1951	0.02946*** (0.01120)	− 0.01025 (0.01348)	0.00781 (0.02102)	0.02770 (0.03934)	− 0.07101 (0.10787)	0.39952 (0.31197)
1952–1956	0.03204** (0.01287)	0.00121 (0.01535)	0.03411 (0.02390)	0.04976 (0.04503)	− 0.05725 (0.12627)	0.50737 (0.36914)
1957–1961	0.04012*** (0.01472)	0.00981 (0.01741)	0.01710 (0.02709)	0.05658 (0.05117)	− 0.08386 (0.14508)	0.55745 (0.42550)
1962–1966	0.04199** (0.01680)	− 0.00935 (0.01976)	− 0.02409 (0.03074)	0.06873 (0.05848)	− 0.12830 (0.16687)	0.66361 (0.49525)
1967–1971	0.00925 (0.01931)	− 0.05410** (0.02269)	− 0.08208** (0.03507)	0.02033 (0.06735)	− 0.12613 (0.19746)	1.11543** (0.56176)
1972–1976	− 0.11520*** (0.02523)	− 0.21855*** (0.03127)	− 0.17663*** (0.04688)	0.04970 (0.09104)	− 0.47756** (0.24356)	0.30805 (0.64109)
1977–1981	− 0.21026*** (0.06747)	− 0.34801*** (0.11585)	− 0.07815 (0.09967)	0.11513 (0.08989)		
1982–1988	− 0.86045*** (0.06771)					
Mother’s age at birth						
13–16	0.02824 (0.02549)	− 0.01939 (0.02995)	0.02807 (0.04759)	0.10819 (0.07789)	− 0.15398 (0.20559)	0.00896 (0.44972)
17–18	0.04595*** (0.00798)	0.01947** (0.00954)	0.01550 (0.01476)	0.08991*** (0.02798)	− 0.00266 (0.07461)	0.16348 (0.19943)
19–20	0.03459*** (0.00518)	0.01143* (0.00621)	0.01254 (0.00955)	0.03935** (0.01847)	0.00471 (0.05320)	0.02776 (0.16311)
21–22	0.02259*** (0.00386)	0.00655 (0.00455)	0.00968 (0.00703)	0.03578*** (0.01357)	0.00729 (0.03912)	− 0.13967 (0.12676)
23–24	0.01192*** (0.00301)	0.00530 (0.00354)	0.00180 (0.00548)	0.02330** (0.01064)	− 0.00667 (0.03183)	− 0.13054 (0.10725)
25–26	0	0	0	0	0	0
27–28	− 0.01390*** (0.00301)	0.00115 (0.00349)	− 0.00616 (0.00540)	0.00302 (0.01045)	0.00771 (0.03193)	− 0.12030 (0.10334)
29–30	− 0.02905*** (0.00367)	− 0.00106 (0.00427)	− 0.01162* (0.00660)	− 0.01103 (0.01297)	− 0.03981 (0.03860)	− 0.04998 (0.11436)
31–32	− 0.03629*** (0.00468)	− 0.00659 (0.00540)	− 0.02440*** (0.00835)	− 0.01573 (0.01620)	− 0.00657 (0.04749)	− 0.26993* (0.14177)
33–34	− 0.05320*** (0.00571)	− 0.00459 (0.00660)	− 0.02019** (0.01018)	− 0.03093 (0.01993)	0.02278 (0.05777)	− 0.23611 (0.18171)
35–36	− 0.06854*** (0.00685)	− 0.00573 (0.00790)	− 0.03538*** (0.01220)	− 0.04967** (0.02377)	− 0.02771 (0.06881)	− 0.28327 (0.19572)
37–38	− 0.07960*** (0.00816)	− 0.02039** (0.00947)	− 0.03885*** (0.01457)	− 0.06870** (0.02802)	− 0.05381 (0.08092)	− 0.37651 (0.24322)
39–40	− 0.10099*** (0.00958)	− 0.02028* (0.01103)	− 0.02595 (0.01698)	− 0.08381** (0.03266)	0.00826 (0.09745)	− 0.15673 (0.28714)
41–42	− 0.11485*** (0.01183)	− 0.02197 (0.01347)	− 0.03145 (0.02091)	− 0.09401** (0.03987)	− 0.13450 (0.11481)	− 0.65669** (0.32159)
43–44	− 0.12348*** (0.01556)	− 0.01922 (0.01822)	− 0.06556** (0.02732)	− 0.12882** (0.05301)	− 0.19230 (0.11969)	− 0.81543 (0.55513)
45–46	− 0.16532*** (0.02555)	− 0.01505 (0.02839)	0.00264 (0.04325)	− 0.06443 (0.08183)	0.17108 (0.21159)	− 0.27840 (0.29998)
47–48	− 0.15807*** (0.05316)	0.07733 (0.05860)	− 0.11308 (0.09448)	0.04497 (0.22270)	0.55607 (0.40251)	0.17700 (0.50043)
49–53	− 0.16718 (0.16920)	0.06967 (0.19652)	0.18327 (0.24877)	0.29115 (0.24706)		
Constant	0.78815*** (0.01159)	0.83388*** (0.01387)	0.42771*** (0.02162)	0.24628*** (0.04042)	0.33882*** (0.11133)	− 0.03009 (0.32316)
Observations	620,203	498,139	412,787	170,908	46,601	12,428
*R*-squared	0.00736	0.00232	0.00444	0.00368	0.01156	0.07527
Number of biosiblingsetid	374,489	327,667	288,340	141,882	42,995	11,937

Swedish register data, authors’ own calculations

Robust standard errors in parentheses

****p* < 0.01; ***p* < 0.05; **p* < 0.1

**Table 5:** Linear probability models for parity transitions, sibling fixed effects, robust standard errors, Swedish women born in 1932–1988

Variables	Women					
	Transition 0–1	Transition 1–2	Transition 2–3	Transition 3–4	Transition 4–5	Transition 5–6
Birth order						
1	0	0	0	0	0	0
2	0.00126 (0.00184)	− 0.00892*** (0.00226)	− 0.01804*** (0.00350)	− 0.01876*** (0.00685)	− 0.04897** (0.02075)	− 0.00933 (0.05825)
3	0.00170 (0.00300)	− 0.01385*** (0.00368)	− 0.03422*** (0.00568)	− 0.02838*** (0.01074)	− 0.01297 (0.03070)	− 0.08772 (0.07613)
4	0.00170 (0.00413)	− 0.01756*** (0.00510)	− 0.03682*** (0.00788)	− 0.03829*** (0.01462)	− 0.05050 (0.04201)	− 0.14342 (0.10151)
5	0.00528 (0.00539)	− 0.02219*** (0.00664)	− 0.04544*** (0.01028)	− 0.04479** (0.01870)	− 0.04013 (0.05356)	− 0.14767 (0.12889)
6	0.01082 (0.00687)	− 0.02755*** (0.00838)	− 0.05561*** (0.01313)	− 0.05861** (0.02376)	− 0.08619 (0.06207)	− 0.07477 (0.15910)
7	− 0.00108 (0.00930)	− 0.03620*** (0.01126)	− 0.05814*** (0.01706)	− 0.01841 (0.02959)	− 0.13598* (0.07826)	0.05657 (0.16823)
8	0.00615 (0.01227)	− 0.03871*** (0.01498)	− 0.04292* (0.02306)	− 0.05038 (0.03785)	0.02547 (0.08828)	− 0.34641* (0.17681)
9	0.02954* (0.01558)	− 0.05983*** (0.01901)	− 0.01605 (0.03046)	− 0.11344** (0.04989)	− 0.06182 (0.10492)	0.22585 (0.30320)
10	0.00219 (0.01926)	− 0.07385*** (0.02370)	− 0.12572*** (0.03413)	− 0.00174 (0.05862)	− 0.45245*** (0.11803)	0.18380 (0.27834)
Year of birth						
1932–1936	0	0	0	0	0	0
1937–1941	0.00912 (0.00743)	0.00266 (0.01060)	− 0.03822** (0.01646)	− 0.02414 (0.03061)	0.04700 (0.07893)	− 0.15302 (0.18887)
1942–1946	0.02629*** (0.00822)	0.00335 (0.01145)	− 0.06108*** (0.01778)	− 0.07645** (0.03331)	0.00998 (0.09045)	0.01390 (0.22146)
1947–1951	0.02732*** (0.00937)	0.00594 (0.01276)	− 0.03840* (0.01979)	− 0.07121* (0.03741)	0.00481 (0.10314)	0.09364 (0.26533)
1952–1956	0.02086* (0.01085)	0.01670 (0.01449)	0.00291 (0.02245)	− 0.04573 (0.04264)	0.01002 (0.11991)	0.23133 (0.31713)
1957–1961	0.02581** (0.01252)	0.02997* (0.01642)	0.01842 (0.02545)	− 0.05186 (0.04862)	0.04774 (0.13845)	0.29070 (0.37688)
1962–1966	0.02921** (0.01440)	0.02477 (0.01861)	− 0.02455 (0.02884)	− 0.07906 (0.05527)	− 0.00611 (0.16014)	0.46757 (0.44156)
1967–1971	0.01282 (0.01659)	− 0.00122 (0.02118)	− 0.06916** (0.03271)	− 0.15133** (0.06299)	− 0.01127 (0.18494)	0.56982 (0.50517)
1972–1976	− 0.08939*** (0.02254)	− 0.10975*** (0.02796)	− 0.18020*** (0.04205)	− 0.16224* (0.08615)	0.10412 (0.21988)	− 0.04773 (0.59381)
1977–1981	− 0.37642*** (0.06708)	− 0.50276*** (0.09866)	− 0.06803 (0.17224)	− 0.28685 (0.37505)	− 0.94245*** (0.24594)	
1982–1988	− 0.50035*** (0.17304)	0.12925* (0.07459)	− 0.52120*** (0.10381)			
Mother’s age at birth						
13–16	0.05508** (0.02354)	− 0.01720 (0.03193)	− 0.00494 (0.04879)	− 0.03253 (0.08651)	0.32328 (0.22733)	− 0.27430 (0.49228)
17–18	0.03100*** (0.00666)	− 0.01567* (0.00896)	0.00180 (0.01371)	0.01642 (0.02665)	0.03826 (0.07354)	− 0.22663 (0.20299)
19–20	0.02260*** (0.00451)	0.00189 (0.00577)	− 0.00297 (0.00894)	0.00167 (0.01755)	− 0.01007 (0.05184)	− 0.01271 (0.12678)
21–22	0.01438*** (0.00331)	0.00123 (0.00424)	− 0.00346 (0.00656)	0.01039 (0.01271)	0.02659 (0.03765)	0.05854 (0.10058)
23–24	0.00702*** (0.00263)	− 0.00295 (0.00332)	0.00091 (0.00513)	0.00985 (0.00998)	− 0.00628 (0.02947)	− 0.01793 (0.08692)
25–26	0	0	0	0	0	0
27–28	− 0.00547** (0.00266)	− 0.00053 (0.00329)	− 0.00614 (0.00509)	− 0.00033 (0.01000)	− 0.01596 (0.03011)	− 0.00403 (0.09402)
29–30	− 0.01227*** (0.00321)	− 0.00041 (0.00400)	− 0.01171* (0.00616)	0.00269 (0.01212)	0.05728 (0.03690)	− 0.15662 (0.10849)
31–32	− 0.01489*** (0.00410)	0.00388 (0.00507)	− 0.01317* (0.00781)	0.00140 (0.01518)	0.00043 (0.04577)	− 0.00200 (0.14679)
33–34	− 0.02384*** (0.00501)	0.00335 (0.00619)	− 0.03010*** (0.00955)	− 0.01108 (0.01879)	0.00175 (0.05618)	− 0.04044 (0.16170)
35–36	− 0.03064*** (0.00595)	− 0.00542 (0.00737)	− 0.02060* (0.01135)	0.00391 (0.02213)	0.02299 (0.06459)	− 0.12322 (0.18860)
37–38	− 0.04168*** (0.00714)	− 0.00641 (0.00877)	− 0.04193*** (0.01351)	− 0.02758 (0.02620)	− 0.03038 (0.07979)	− 0.32341 (0.23530)
39–40	− 0.05027*** (0.00840)	0.00902 (0.01022)	− 0.03234** (0.01582)	− 0.00740 (0.03015)	0.05269 (0.08929)	− 0.18406 (0.23766)
41–42	− 0.06698*** (0.01033)	0.00322 (0.01247)	− 0.01672 (0.01919)	− 0.01341 (0.03716)	− 0.05502 (0.10959)	− 0.19373 (0.32482)
43–44	− 0.06391*** (0.01381)	0.00062 (0.01611)	− 0.05875** (0.02470)	0.00209 (0.04650)	0.11989 (0.12966)	− 0.11122 (0.37300)
45–46	− 0.09481*** (0.02381)	0.04303* (0.02553)	− 0.03872 (0.03927)	− 0.03249 (0.07405)	− 0.22127 (0.16456)	0.18121 (0.47726)
47–48	− 0.06465 (0.05218)	− 0.08060 (0.07017)	0.04344 (0.09618)	0.21559 (0.16936)	− 0.59108** (0.27856)	
49–53	− 0.04526 (0.04656)	0.07006 (0.16031)	− 0.03080 (0.30222)	− 0.37695*** (0.04907)		
Constant	0.85434*** (0.00971)	0.83571*** (0.01311)	0.44487*** (0.02040)	0.31848*** (0.03852)	0.23934** (0.10670)	0.20802 (0.27535)
Observations	592,677	516,527	434,435	172,277	41,737	9945
*R*-squared	0.00409	0.00143	0.00493	0.00646	0.01771	0.07452
Number of biosiblingsetid	365,730	335,356	298,507	140,926	38,055	9440

Swedish register data, authors’ own calculations

Robust standard errors in parentheses

****p* < 0.01; ***p* < 0.05; **p* < 0.1

**Table 6:** OLS regression on number of children by number of additional siblings, stratified by birth order, Swedish men born in 1932–1988

Variables	Men					
	Birth order 1	Birth order 2	Birth order 3	Birth order 4	Birth order 5	Birth order 6
Number of additional siblings						
0	0	0	0	0	0	0
1	0.11104*** (0.00584)	0.08097*** (0.00622)	0.07385*** (0.00995)	0.02716 (0.01707)	0.04579 (0.02837)	0.07232 (0.04516)
2	0.17466*** (0.00690)	0.12279*** (0.00900)	0.11950*** (0.01520)	0.08027*** (0.02533)	0.09769** (0.04072)	0.07997 (0.06283)
3	0.23590*** (0.00932)	0.15680*** (0.01404)	0.13888*** (0.02290)	0.09428** (0.03702)	0.03996 (0.05857)	0.12526 (0.09029)
4	0.27757*** (0.01419)	0.23939*** (0.02161)	0.13939*** (0.03444)	0.13718** (0.05401)	0.16745** (0.08234)	0.23747* (0.12784)
5	0.32605*** (0.02169)	0.23117*** (0.03280)	0.23778*** (0.05038)	0.10940 (0.07862)	0.23684** (0.11978)	0.13608 (0.19380)
6	0.28699*** (0.03243)	0.33132*** (0.04807)	0.13826* (0.07339)	0.02704 (0.11125)	− 0.36419** (0.17684)	0.17462 (0.22089)
7	0.36744*** (0.04836)	0.14987** (0.07297)	0.48276*** (0.10752)	0.25893 (0.16953)	0.28951 (0.21449)	0.81839** (0.38413)
8	0.34179*** (0.07359)	0.09310 (0.10118)	− 0.08013 (0.15394)	− 0.11929 (0.20070)	− 0.09060 (0.33648)	0.65235 (0.48717)
9	0.51207*** (0.11220)	0.24047 (0.15956)	0.25619 (0.20944)	− 0.10572 (0.28995)	0.90204* (0.47628)	1.05860 (1.36301)
10	0.36949** (0.17032)	0.51042*** (0.19126)	0.48839* (0.27402)	0.59984 (0.48998)	− 0.00781 (1.33714)	− 0.07049 (0.79853)
11	0.37695 (0.23202)	1.14119*** (0.31611)	− 0.22996 (0.63153)	− 1.23841* (0.64763)	1.12163 (1.34086)	
12	0.22588 (0.28933)	0.72447 (0.49974)	0.52263 (0.88707)	− 1.95840 (1.29300)		
13	− 0.72997 (0.46394)	− 1.90973 (1.22348)	1.02970 (1.25448)	4.04312*** (0.91416)		
14	− 0.83345 (0.86778)		1.03554 (0.88707)			
15	− 0.89149 (0.86780)	0.05563 (1.22363)				
16	− 1.93002 (1.22736)					
Year of birth						
1932–1936	0	0	0	0	0	0
1937–1941	− 0.01954 (0.02084)	− 0.06438 (0.06480)	0.02278 (0.27984)			
1942–1946	− 0.05590*** (0.02027)	− 0.07770 (0.06396)	− 0.02550 (0.27881)	− 0.04352 (0.08316)	− 0.04993 (0.19105)	− 0.04781 (0.63050)
1947–1951	− 0.06026*** (0.02024)	− 0.08882 (0.06385)	− 0.02526 (0.27872)	− 0.02294 (0.08198)	− 0.03557 (0.18889)	0.07525 (0.62577)
1952–1956	− 0.08051*** (0.02042)	− 0.09119 (0.06389)	− 0.01547 (0.27873)	− 0.00176 (0.08190)	− 0.03356 (0.18893)	0.07277 (0.62588)
1957–1961	− 0.14786*** (0.02112)	− 0.15616** (0.06407)	− 0.08710 (0.27882)	− 0.06044 (0.08232)	− 0.08797 (0.18933)	0.07619 (0.62607)
1962–1966	− 0.25177*** (0.02328)	− 0.26583*** (0.06437)	− 0.18217 (0.27890)	− 0.15686* (0.08321)	− 0.21893 (0.19052)	− 0.05599 (0.62704)
1967–1971	− 0.52710*** (0.03267)	− 0.50947*** (0.06611)	− 0.42965 (0.27926)	− 0.41736*** (0.08545)	− 0.47519** (0.19314)	− 0.22417 (0.62906)
1972–1976	− 0.83883*** (0.06718)	− 0.91845*** (0.08161)	− 0.90720*** (0.28273)	− 0.85090*** (0.10142)	− 0.91254*** (0.20874)	− 0.65174 (0.63909)
1977–1981	− 1.30414*** (0.29562)	− 1.43556*** (0.28423)	− 1.36931*** (0.37364)	− 1.31454*** (0.25415)	− 1.40804*** (0.39862)	− 1.19502 (0.77592)
1982–1988		− 2.00947 (1.49983)		− 1.51149* (0.79014)		
Mother’s age at birth						
13–16	0.22282*** (0.05859)	− 1.97915 (1.22346)				
17–18	0.13702*** (0.01448)	0.16475** (0.06399)	− 0.05960 (0.41962)			
19–20	0.09904*** (0.00922)	0.06357*** (0.01998)	− 0.03267 (0.07190)	0.48881 (0.31477)	− 2.08971 (1.34949)	
21–22	0.06002*** (0.00804)	0.04225*** (0.01241)	0.06465** (0.02957)	0.00822 (0.08362)	0.54092 (0.35054)	
23–24	0.02467*** (0.00768)	0.03005*** (0.01025)	0.03029 (0.02071)	0.04851 (0.04690)	− 0.12145 (0.11143)	− 0.08835 (0.34451)
25–26	0	0	0	0	0	0
27–28	− 0.03248*** (0.00813)	− 0.00287 (0.00925)	− 0.00532 (0.01677)	0.02856 (0.03334)	− 0.00838 (0.06672)	0.23909* (0.13810)
29–30	− 0.07661*** (0.00921)	− 0.04373*** (0.00952)	− 0.01609 (0.01652)	0.00408 (0.03254)	− 0.07541 (0.06386)	0.13315 (0.12862)
31–32	− 0.12174*** (0.01105)	− 0.07660*** (0.01027)	− 0.04768*** (0.01687)	− 0.04443 (0.03255)	− 0.09074 (0.06415)	0.12893 (0.12611)
33–34	− 0.16321*** (0.01375)	− 0.10752*** (0.01152)	− 0.06154*** (0.01761)	− 0.04688 (0.03330)	− 0.05422 (0.06401)	0.20862 (0.12711)
35–36	− 0.17536*** (0.01723)	− 0.15801*** (0.01358)	− 0.07034*** (0.01883)	− 0.05639 (0.03433)	− 0.10482 (0.06519)	0.08848 (0.12776)
37–38	− 0.21366*** (0.02172)	− 0.16686*** (0.01703)	− 0.11897*** (0.02097)	− 0.11171*** (0.03609)	− 0.15039** (0.06738)	0.14309 (0.13056)
39–40	− 0.22268*** (0.02952)	− 0.24165*** (0.02258)	− 0.14495*** (0.02479)	− 0.14624*** (0.03917)	− 0.16113** (0.07034)	0.08552 (0.13339)
41–42	− 0.25879*** (0.04272)	− 0.25366*** (0.03377)	− 0.14913*** (0.03356)	− 0.14314*** (0.04650)	− 0.17267** (0.07751)	− 0.10323 (0.14057)
43–44	− 0.27440*** (0.07151)	− 0.30691*** (0.05934)	− 0.30146*** (0.05444)	− 0.13525** (0.06229)	− 0.25841*** (0.09381)	− 0.03169 (0.15536)
45–46	− 0.23821* (0.14289)	− 0.25051** (0.12185)	− 0.44273*** (0.11195)	− 0.19400* (0.11251)	− 0.01306 (0.14914)	0.14074 (0.20966)
47–48	− 0.60240 (0.40948)	− 0.12465 (0.30659)	− 0.47382** (0.23428)	− 0.45883* (0.24882)	0.01068 (0.31415)	0.46232 (0.37512)
49–53	1.38997 (0.86848)	0.06429 (0.86529)	2.49851*** (0.88709)	0.19493 (0.57905)	− 0.20269 (0.67131)	
Constant	1.79300*** (0.02108)	1.94518*** (0.06416)	1.93114*** (0.27899)	1.97033*** (0.08246)	2.04975*** (0.18718)	1.73206*** (0.62032)
Observations	302,475	226,331	102,580	39,297	14,834	5999
*R*-squared	0.02044	0.01931	0.02079	0.02139	0.02474	0.02216

Swedish register data, authors’ own calculations

Standard errors in parentheses

****p* < 0.01; ***p* < 0.05; **p* < 0.1

**Table 7:** OLS regression on number of children by number of additional siblings, stratified by birth order, Swedish women born in 1932–1988

Variables	Women					
	Birth order 1	Birth order 2	Birth order 3	Birth order 4	Birth order 5	Birth order 6
Number of additional siblings						
0	0	0	0	0	0	0
1	0.12054*** (0.00542)	0.09529*** (0.00580)	0.08757*** (0.00927)	0.09476*** (0.01572)	0.11185*** (0.02624)	0.05926 (0.04179)
2	0.21763*** (0.00642)	0.17981*** (0.00838)	0.15933*** (0.01420)	0.16653*** (0.02310)	0.22657*** (0.03714)	0.04528 (0.05748)
3	0.29617*** (0.00867)	0.24664*** (0.01299)	0.22592*** (0.02131)	0.19612*** (0.03363)	0.12904** (0.05240)	0.26374*** (0.07931)
4	0.35810*** (0.01301)	0.28717*** (0.02000)	0.19530*** (0.03155)	0.26741*** (0.04901)	0.11251 (0.07610)	0.16138 (0.11517)
5	0.40121*** (0.01986)	0.36001*** (0.03017)	0.26925*** (0.04744)	0.21856*** (0.07009)	0.33227*** (0.10930)	0.34468** (0.16645)
6	0.41376*** (0.03027)	0.33357*** (0.04566)	0.35092*** (0.06955)	0.24197** (0.10836)	0.44176*** (0.16505)	0.42537** (0.20779)
7	0.52222*** (0.04542)	0.42660*** (0.06486)	0.44699*** (0.10341)	0.09181 (0.15927)	0.33230* (0.19683)	− 0.30565 (0.26911)
8	0.57188*** (0.06455)	0.52323*** (0.10607)	0.30183* (0.16573)	− 0.07570 (0.19898)	0.39970 (0.29534)	0.89393 (0.56941)
9	0.62460*** (0.09340)	0.54847*** (0.15122)	0.45993*** (0.17728)	0.19583 (0.31372)	1.17910** (0.54603)	0.54552 (0.62574)
10	0.90336*** (0.14337)	0.34134* (0.18024)	0.62405** (0.29606)	− 0.50006 (0.52461)	0.55348 (0.61291)	− 0.21749 (1.24546)
11	0.69865*** (0.16159)	0.03023 (0.24822)	0.28336 (0.38223)	− 0.06090 (1.17054)	− 0.44287 (0.70537)	0.25316 (0.88076)
12	0.24044 (0.28892)	0.15820 (0.41954)	1.95036*** (0.66133)	1.08808 (0.69078)	0.37006 (0.85669)	
13	0.44864 (0.45684)	− 0.21292 (0.55545)	1.23739* (0.66168)			
14	0.07889 (0.64601)	0.25579 (0.55491)				
15	2.60496*** (0.79108)					
16	0.13929 (1.11874)					
Year of birth						
1932–1936	0	0	0	0	0	0
1937–1941	− 0.06010*** (0.01872)	0.04217 (0.05629)	− 0.19291 (0.22053)			
1942–1946	− 0.08528*** (0.01822)	− 0.01514 (0.05545)	− 0.20947 (0.21930)	− 0.07379 (0.07831)	0.26519 (0.19400)	− 1.26409** (0.52966)
1947–1951	− 0.06570*** (0.01817)	− 0.01497 (0.05536)	− 0.19449 (0.21922)	− 0.07742 (0.07724)	0.23795 (0.19184)	− 1.25255** (0.52750)
1952–1956	− 0.03733** (0.01835)	0.02125 (0.05539)	− 0.17430 (0.21922)	− 0.05339 (0.07724)	0.24846 (0.19175)	− 1.25564** (0.52722)
1957–1961	− 0.03356* (0.01903)	0.02346 (0.05557)	− 0.14726 (0.21932)	0.01622 (0.07758)	0.35829* (0.19205)	− 1.17480** (0.52737)
1962–1966	− 0.13368*** (0.02109)	− 0.05029 (0.05588)	− 0.21140 (0.21941)	− 0.04112 (0.07840)	0.33666* (0.19300)	− 1.19124** (0.52835)
1967–1971	− 0.29178*** (0.02989)	− 0.22381*** (0.05765)	− 0.37366* (0.21983)	− 0.17787** (0.08046)	0.16175 (0.19512)	− 1.24174** (0.53021)
1972–1976	− 0.71587*** (0.06430)	− 0.62976*** (0.07273)	− 0.83695*** (0.22407)	− 0.56247*** (0.09452)	− 0.26369 (0.20850)	− 1.74190*** (0.53989)
1977–1981	− 1.22904*** (0.23167)	− 1.33660*** (0.22876)	− 1.46089*** (0.31281)	− 1.57110*** (0.26474)	− 1.30471*** (0.36001)	− 2.35581*** (0.67441)
1982–1988				− 1.96300** (0.86812)	− 2.36741* (1.36887)	
Mother’s age at birth						
13–16	0.16360*** (0.05407)	0.08192 (0.45348)				
17–18	0.12852*** (0.01337)	0.09176 (0.05915)	0.00678 (0.51244)	− 1.06090 (1.17054)		
19–20	0.09765*** (0.00855)	0.07124*** (0.01819)	0.13388* (0.07035)	− 0.47639 (0.42361)		
21–22	0.06295*** (0.00749)	0.04027*** (0.01148)	0.05720** (0.02714)	0.07439 (0.08020)	− 0.00908 (0.26634)	
23–24	0.03375*** (0.00717)	0.03023*** (0.00951)	0.02200 (0.01930)	0.01857 (0.04213)	− 0.01545 (0.10305)	− 0.43627 (0.27250)
25–26	0	0	0	0	0	0
27–28	− 0.02634*** (0.00759)	− 0.01315 (0.00860)	− 0.05027*** (0.01567)	− 0.05620* (0.03021)	− 0.01003 (0.06225)	0.02894 (0.13049)
29–30	− 0.06363*** (0.00856)	− 0.03539*** (0.00882)	− 0.03324** (0.01541)	− 0.04307 (0.02943)	− 0.03144 (0.05933)	− 0.10125 (0.12370)
31–32	− 0.07960*** (0.01029)	− 0.07473*** (0.00957)	− 0.05144*** (0.01573)	− 0.05980** (0.02960)	− 0.07779 (0.05944)	0.02143 (0.12195)
33–34	− 0.09959*** (0.01285)	− 0.08644*** (0.01071)	− 0.08862*** (0.01646)	− 0.11193*** (0.03018)	− 0.10879* (0.05990)	− 0.13980 (0.12251)
35–36	− 0.12823*** (0.01604)	− 0.12019*** (0.01270)	− 0.10230*** (0.01756)	− 0.08933*** (0.03114)	− 0.02967 (0.06103)	− 0.14732 (0.12340)
37–38	− 0.17065*** (0.02059)	− 0.15089*** (0.01575)	− 0.13246*** (0.01970)	− 0.14361*** (0.03281)	− 0.05720 (0.06291)	− 0.20938* (0.12490)
39–40	− 0.13603*** (0.02753)	− 0.15835*** (0.02098)	− 0.14439*** (0.02343)	− 0.14580*** (0.03580)	− 0.12269* (0.06600)	− 0.12874 (0.12833)
41–42	− 0.11907*** (0.03949)	− 0.21463*** (0.03096)	− 0.16209*** (0.03056)	− 0.19230*** (0.04272)	− 0.10763 (0.07186)	− 0.13630 (0.13354)
43–44	− 0.23447*** (0.06576)	− 0.15002*** (0.05298)	− 0.24891*** (0.04977)	− 0.16920*** (0.05920)	− 0.21174** (0.08731)	− 0.12517 (0.15006)
45–46	− 0.15811 (0.14070)	− 0.14114 (0.11686)	− 0.16800* (0.09397)	− 0.11161 (0.10046)	− 0.18692 (0.14068)	− 0.36385* (0.19875)
47–48	− 0.63943 (0.42315)	− 0.51161 (0.33540)	− 0.40210* (0.21025)	− 0.17532 (0.25252)	0.52236 (0.34435)	− 0.33699 (0.32711)
49–53					0.53350 (0.61283)	0.80065 (1.24949)
Constant	1.86346*** (0.01897)	1.92243*** (0.05567)	2.20813*** (0.21953)	2.13832*** (0.07747)	1.83391*** (0.18980)	3.44110*** (0.52003)
Observations	290,606	215,604	97,707	37,721	14,335	5911
*R*-squared	0.01938	0.01607	0.01499	0.01480	0.01689	0.01839

Swedish register data, authors’ own calculations

Standard errors in parentheses

****p* < 0.01; ***p* < 0.05; **p* < 0.1
